# Rapid Ferric Transformation by Reductive Dissolution of Schwertmannite for Highly Efficient Catalytic Degradation of Rhodamine B

**DOI:** 10.3390/ma11071165

**Published:** 2018-07-09

**Authors:** Jingyu Ran, Bo Yu

**Affiliations:** 1 School of Chemical Engineering, Guizhou Institute of Technology, Guiyang 550003, China; 2 State Key Laboratory of Chemical Engineering, School of Chemical Engineering and Technology, Tianjin University, Tianjin 300072, China; tjuyubo@163.com

**Keywords:** ferric transformation, reductive dissolution, schwertmannite, heterogeneous

## Abstract

In this study, reductive dissolution of iron oxides was considered for the acceleration of the transformation from Fe(III) to Fe(II) to improve the degradation of rhodamine B (RhB) by potassium persulfate (PS) activation on schwertmannite. The addition of hydroxylamine (HA) showed an enhancement effect on the degradation at pH 3 and 5, but insignificant efficiency of the addition was obtained at pH 9. The surface reduction from Fe(III)-OH to Fe(II)-OH by HA was considered dominant for the acceleration of PS activation through the reductive dissolution process, and the hydroxyl and sulfate radicals generated by the decomposition of surface complexes were main primary reactive oxidants that contributed to the degradation of RhB.

## 1. Introduction

Advanced oxidation processes (AOPs) are considered efficient for the abiotic degradation of organic pollutants in water treatment due to the high oxidation activity of radicals towards organics, especially biological toxic and non-degradable organics, which are difficult to be degraded by conventional or biological oxidation methods [1]. Recently, sulfate radicals (${\mathrm{SO}}_{4}^{•-}$) based AOPs has attracted considerable attention due to the high standard redox potential of ${\mathrm{SO}}_{4}^{•-}$ (2.6 V) [2], which is comparable to that of hydroxyl radicals (•OH, 2.8 V) [3]. As the important way to generate ${\mathrm{SO}}_{4}^{•-}$, the activation of peroxymonosulfate (PMS) and persulfate (PS) has been widely studied via heating, UV irradiation and transition metals activated [4,5,6,7,8,9,10,11,12]. PS or PMS activation can also be achieved by transition metals [4,5,11]. Considering the environmental friendly nature and the advantages of cost effectiveness, Fe(II) has always been selected as the activator of PS or PMS for the generation of ${\mathrm{SO}}_{4}^{•-}$ [13,14,15]. However, Fe(II)/PS process shows slow transformation from Fe(III) to Fe(II), which further results in low efficiency for PS or PMS activation and radicals generation [11,15]. Although many efforts have been made to accelerate the transformation from Fe(III) to Fe(II) by employing UV irradiation [16], electrochemistry [17] and reducing agents [15,18], the accumulation of ferric oxide sludge and acidic pH conditions still limit the widespread application [19]. To avoid the accumulation of ferric oxide sludge and extend the condition to neutral pH, iron oxides such as ferrihydrite, lepidocrocite, goethite and hematite have been widely investigated as catalysts for heterogeneous AOPs [20,21,22,23,24]. However, the outer Fe(III) layer formed by the oxidation on iron oxides will passivate the surface of catalysts and inhibit the catalytic efficiency toward AOPs. The slow transformation of Fe(III) to Fe(II) still limits the peroxide or persulfate activation and radical generation.

Schwertmannite (Sch) is a poorly crystalline Fe(III)-oxyhydroxysulfate mineral, which always precipitates in sulfate-rich acid mine drainage (AMD). It is always represented as Fe_8_O_8_(OH)_8−2*x*_(SO_4_)*_x_*, where *x* varies from 1 to 1.75 [25,26]. Reductive dissolution is a dissolution process of ferric minerals with the release of Fe(II) from Fe(III) oxides to aqueous solutions. As hydroxylamine (HA) adsorbs onto the surface of iron oxides, surface complex of [Fe(III)-HA] forms followed by electron transfer between Fe(III) and HA, which subsequently results in the formation of Fe(II) and semi-stable state surface complexes. Fe(II) will be directly released through the decomposition of surface complexes. Therefore, addition of HA enhanced the transformation from Fe(III) to Fe(II) on the surface, which results in good efficiency for the improvement of PS activation on schwertmannite. Although schwertmannite has been investigated as a new Fenton-like catalyst in the oxidation of phenol by hydrogen peroxide (H_2_O_2_) [23], to the best of our knowledge, activation of PS on schwertmannite for heterogeneous AOPs has never been reported. Therefore, the objective of this study was to investigate the heterogeneous catalytic oxidation of rhodamine B (RhB) by PS and the effect of reductive dissolution of schwertmannite on the heterogeneous advanced oxidation process.

## 2. Experimental Section

### 2.1. Materials and Chemicals

Ferrous sulfate (FeSO_4_·7H_2_O), ferric chloride (FeCl_3_·6H_2_O), hydrogen peroxide (H_2_O_2_), potassium persulfate (PS), hydroxylamine (HA), rhodamine B (RhB), tert-butyl alcohol (TBA) and methanol were of analytical reagent grade and purchased from Tianjin Guangfu Technology Development Co., Ltd. (Tianjin, China) All chemicals were used without further purification, and Milli-Q water was used throughout this study. 

### 2.2. Synthesis of Schwertmannite

Schwertmannite was synthesized via Fe(II) oxidation method [27]: 16.45 g FeSO_4_·7H_2_O was dissolved in 1 L deionized water and reacted with 5.3 mL 30% H_2_O_2_. The solution became dark red, and a red-orange material precipitated immediately with the final pH of 2.5 after 24 h. The solid was then centrifuged, washed three times and freeze-dried for further use.

### 2.3. Catalytic Oxidation of RhB

All experiments were performed in 150 mL triangular flasks with a constant stirring at 25 °C. Solutions with desired concentrations of RhB (0.5 M), PS (0.5 M) and HA (10 mM) were prepared before the reaction, respectively. In a typical experiment, 50 mg schwertmannite solid was hydrated in deionized water for 1 h at a given pH, which was adjusted to desired values by using 0.5 M HCl or NaOH solution during the equilibration. After that, the prepared RhB, PS and HA solutions was added, and the desired initial pH was adjusted rapidly by using 0.5 M HCl or NaOH solution. After the addition of RhB, PS and HA, the final volume of the solution was 100 mL, and the initial concentrations of iron oxides, RhB, PS and HA were 0.5 g/L, 25 mM, 5 mM and 0–1 mM, respectively. Furthermore, Fe(III) as FeCl_3_ was employed and substituted for schwertmannite to investigate the effect of dissolved iron on the degradation process, the concentration of Fe(III) was 25 µM after mixing. Besides, to understand the activation mechanism of PS on schwertmannite, methanol and TBA were employed as scavengers for radicals formed by the PS activation. After mixing of RhB, PS, and HA, methanol or TBA were added in to the system, and the concentrations were changed from 0 to 1 mM, respectively. During the oxidation experiments, samples were withdrawing regularly and centrifuged for 2 min at 12,000 rpm. The supernatant was then obtained for the determination of RhB and dissolved iron concentrations. All data were determined and collected by dual experiments.

### 2.4. Characterization

Morphology of the synthesized schwertmannite was characterized by field emission scanning electron microscopy (SEM, S4800, Hitachi, Tokyo, Japan) and high-resolution analytical transmission electron microscopy (TEM, Fei Tecnai G2 F20, FEI, Hillsboro, OR, USA). The crystalline structure of schwertmannite was characterized by powder X-ray diffraction pattern (XRD), which was employed by using Cu Ka (1.54) radiation on a D/MAX 2500 X-ray diffractometer at the voltage of 40 kV and current of 100 mA. Samples were gently ground and analyzed over a diffraction-angle (2θ) range of 2–90°, with scanning rate of 8°/min. Concentration of RhB was determined at 550 nm by UV–Vis spectrophotometer (UV-6300, Mapada, Shanghai, China). Dissolved iron was measured by inductively coupled plasma–mass spectrometry (ICP-MS, 7700x, Agilent, Santa Clara, CA, USA). 

### 2.5. Computation Details

Orbital energy analysis of intermediates was achieved by DMol3 module of Materials Studio 8.0, and the highest occupied molecular orbital (HOMO) energy level and lowest unoccupied molecular orbital (LUMO) energy level were studied to evaluate the energy needed for electrons to cross the energy barrier based on the frontier molecular orbital analysis [28].

## 3. Results and Discussion

### 3.1. Characterization of Schwertmannite

**Figure 1:**
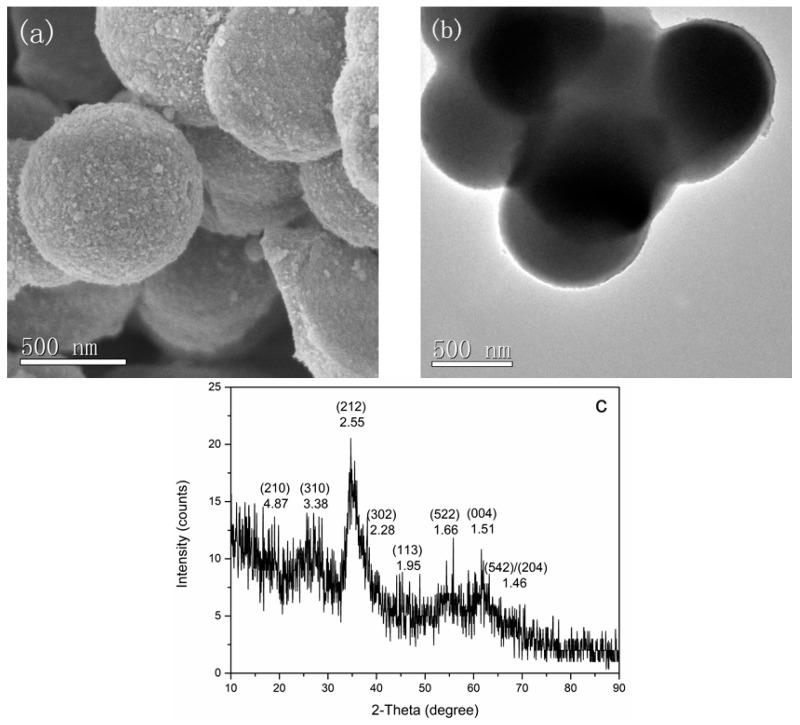
SEM (**a**); TEM (**b**); and XRD (**c**) analysis of synthesized schwertmannite.

Schwertmannite was a Fe(III)-oxyhydroxysulfate oxide with poorly crystalline structure. Morphological analysis achieved by SEM and TEM suggests that the synthesized schwertmannite shows a spherical morphology with the diameter of 500 nm (Figure 1a,b). XRD analysis shows diffraction peaks as (201), (310), (212), (302), (522), (004) and (542)/(204) (Figure 1c), which are in good agreement with schwertmannite (JCPDS, No. 47-1775).

### 3.2. Effect of HA on Degradation of RhB by PS on Schwertmannite

**Figure 2:**
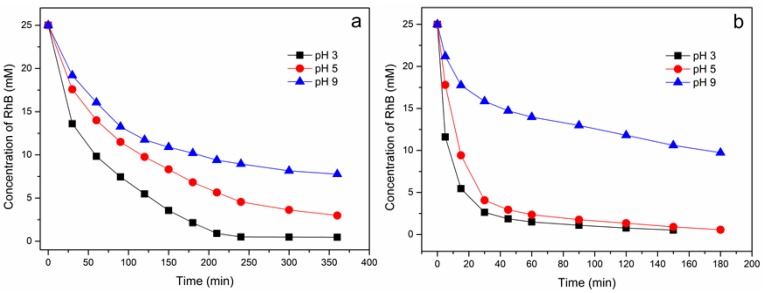
Degradation of RhB in the absence (**a**) and presence (**b**) of HA under various pH conditions. Concentrations: [Schwertmannite] = 0.5 g/L, [RhB] = 25 mM, [PS] = 5 mM, and [HA] = 0.5 mM.

As shown in Figure 2, RhB could be oxidized and degraded by PS activation on schwertmannite at pH 3, 5 and 9, and the degradation of RhB was more efficient in acid conditions. Concentrations of RhB decreased from 25 mM to 0.5, 3.0 and 7.8 mM in 360 min at pH 3, 5 and 9, respectively (Figure 2a). This suggests that schwertmannite shows good catalytic activity for radical generation from PS activation in heterogeneous AOPs. More than 90%, 84% and 37% of RhB was degraded in 30 min with the addition of HA at pH 3, 5 and 9, respectively (Figure 2b). Concentrations of RhB decreased rapidly from 25 mM to 0.5, 0.6 and 9.7 mM in 180 min at pH 3, 5 and 9, respectively. The rapid decreasing concentration of RhB with the addition of HA suggests an enhanced activation of PS by the acceleration of Fe(III)-Fe(II) recycle [15,18], which attributes to the reducing capacity of HA for the transformation from Fe(III) to Fe(II) [29,30].

**Figure 3:**
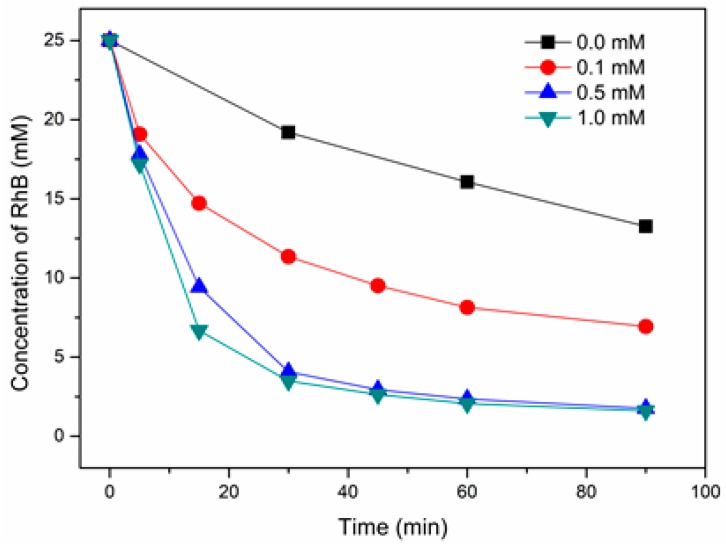
Effect of HA concentration on RhB degradation at pH 5. Concentrations: [Sch] = 0.5 g/L, [RhB] = 25 mM, [PS] = 5 mM, and [HA] = 0–1 mM.

Although HA can accelerate the transformation from Fe(III) to Fe(II) and contribute to the increasing degradation rate of RhB under acid conditions, high concentration of HA will be counterproductive [15]. It was found that the degradation rate of RhB increased rapidly with the increasing concentrations of HA from 0 to 0.5 mM at pH 5, and the concentrations of RhB decreased from 25 mM to 11.5, 7.0 and 1.8 mM in 90 min, respectively (Figure 3). However, the degradation rate of RhB increased slightly when the concentration of HA increased from 0.5 to 1.0 mM (Figure 3). The slightly increased degradation rate of RhB under high HA concentration might be caused by the removal of radicals by excess NH_3_OH^+^ [2,3,15], which is the main form of HA (pKa 5.96 at 25 °C) at pH 3 and 5 in aqueous solutions [31]. Considering the rate constant of 5.0 × 10^8^ M^−1^·s^−1^ for •OH and 1.5 × 10^7^ M^−1^·s^−1^ for ${\mathrm{SO}}_{4}^{•-}$, NH_3_OH^+^ would be the scavenger for •OH and SO radicals, as shown in Equations (1) and (2), when the concentration of NH_3_OH^+^ is high enough [2,3].

(1) $${\mathrm{NH}}_{3}{\mathrm{OH}}^{+}+•\mathrm{OH}\to {\mathrm{OH}}^{-}+\mathrm{nitro}-\mathrm{products}$$

(2) $${\mathrm{NH}}_{3}{\mathrm{OH}}^{+}+{\mathrm{SO}}_{4}^{•-}\to {\mathrm{SO}}_{4}^{2-}+\mathrm{nitro}-\mathrm{products}$$

### 3.3. Release Files of Dissolved Iron from Schwertmannite

**Figure 4:**
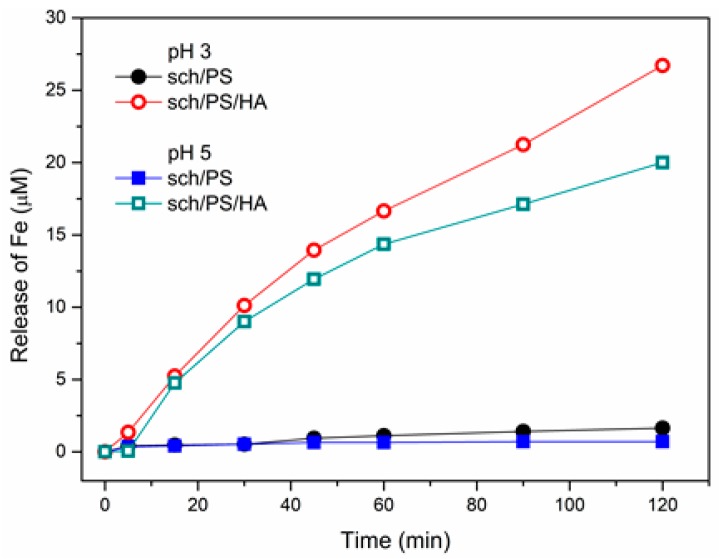
Release files of dissolved iron from schwertmannite during the degradation of RhB under various pH conditions. Concentrations: [Sch] = 0.5 g/L, [RhB] = 25 mM, [PS] = 5 mM, and [HA] = 0.5 mM.

Figure 4 shows the release of dissolved iron from schwertmannite during the degradation of RhB at pH 3 and 5. The dissolved iron was released slowly from schwertmannite without the addition of HA due to the low efficiency of PS activation, and 1.6 and 0.7 µM iron released in 120 min at pH 3 and 5, respectively. However, more Fe(III) was dissolved and released from schwertmannite in the presence of HA during the degradation of RhB. After the addition of HA, 26.7 and 20.0 µM iron was released in 120 min at pH 3 and 5, respectively. Considering the higher degradation rate of RhB in the presence of HA (Figure 2b), the increasing release of dissolved iron indicates the acceleration of transformation from Fe(III) to Fe(II) and the improvement of PS activation on schwertmannite.

### 3.4. Effect of Fe(III) on the Degradation of RhB

**Figure 5:**
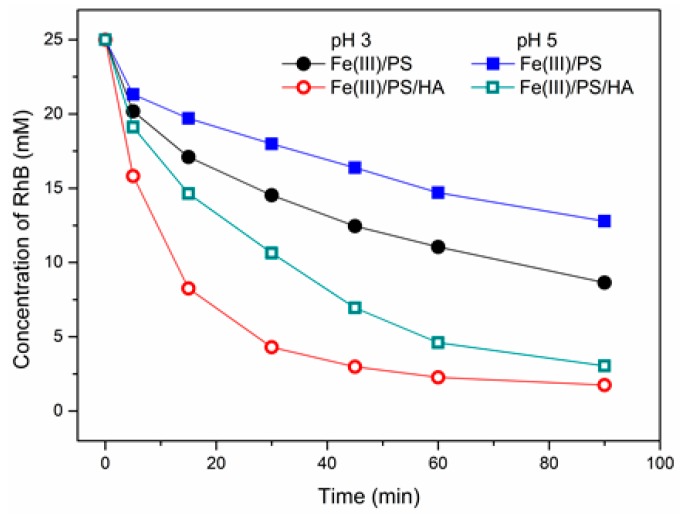
Effect of Fe(III) on RhB degradation under various pH conditions. Homogeneous catalytic degradation of RhB at initial pH 3 and 5. Concentrations: [Fe(III)] = 25 µM, [RhB] = 25 mM, [PS] = 5 mM, and [HA] = 0.5 mM.

To investigate the effect of Fe(III) on the degradation of RhB, ferric chloride (FeCl_3_·6H_2_O) was employed and substituted for schwertmannite during the catalytic process. As shown in Figure 5, under the restriction of PS activation, concentrations of RhB decreased slowly from 25 mM to 8.6 and 12.8 mM in 90 min at pH 3 and 5, respectively (Figure 5). However, it showed higher degradation rate of RhB after the addition of HA: only 1.7 and 3.0 mM RhB remained at 90 min at pH 3 and 5, respectively. The addition of HA contributed to the acceleration of transformation from Fe(III) to Fe(II) due to the reducing capacity, which further improved the generation of radicals from PS activation for the degradation process [15].

### 3.5. Determination of Kinetic Parameters

**Figure 6:**
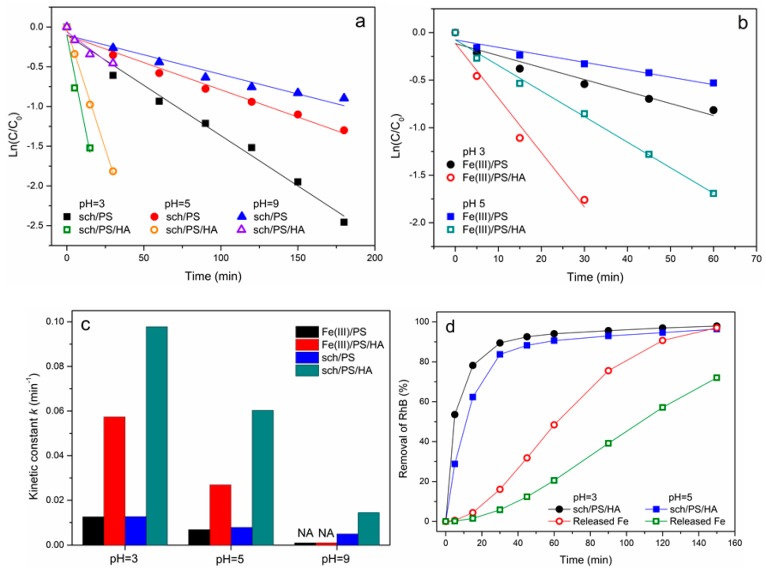
First-order kinetics of RhB degradation in homogeneous and heterogeneous oxidation processes under various pH conditions. Initial RhB degradation rates in: (**a**) Sch/PS and Sch/PS/HA; and (**b**) Fe(III)/PS and Fe(III)/PS/HA. (**c**) Effect of HA on initial RhB degradation rates. (**d**) Effect of released iron by the reductive dissolution during the heterogeneous oxidation process.

The initial kinetic constants of RhB degradation were quite different in homogeneous and heterogeneous processed (Figure 6). The initial kinetic constants were obtained by first-order kinetic equation (Equation (3)), which can also be expressed as Equation (4):

*dC*/*dt* = −*k*_1_*C* (3)

ln(*C*/*C*_0_) = −*kt* (4)

where *C* is the concentration of RhB (mM); *C*_0_ is the initial concentration of RhB (mM); *t* is time (min); and *k* is the initial kinetic constant of RhB degradation (min^−1^). The linear relationship between ln(*C*/*C*_0_) and *t* in heterogeneous and homogeneous process are shown in Figure 6a,b, and the absolute value of linear slope represents the initial kinetic constant *k*. As shown in Figure 6c, the initial kinetic constants are 1.3 × 10^−2^ and 7.8 × 10^−3^ min^−1^ for heterogeneous process and 1.3 × 10^−2^ and 6.9 × 10^−3^ min^−1^ for homogeneous process without the addition of HA at pH 3 and 5, respectively. However, they are 9.8 × 10^−2^ and 6.0 × 10^−2^ min^−1^ for heterogeneous process and 5.7 × 10^−2^ and 2.7 × 10^−2^ min^−1^ for homogeneous process in the presence of HA at pH 3 and 5, respectively. Compared with the homogeneous process, more significant improvement of the initial kinetic constant at pH 3 and 5 in heterogeneous process suggests that the activation of PS enhanced by HA is mainly achieved through heterogeneous catalysis at acid conditions despite the existence of homogeneous process. The homogeneous kinetics and iron releasing kinetics can be described as Equations (5) and (6):

*dC*/*dt* = −*k*_1_*C*·*m* (5)

*dm*/*dt* = −*k*_2_(m* − *m*)^*r*^ (6)

where *m* is the concentration of released iron (µM); m* is the maximum of released iron concentrations (µM); *k*_1_ and *k*_2_ are kinetic constants (min^−1^); and *r* is the activity of iron oxides in the reductive dissolution process. As shown in Figure 6d, degradations were 16.1% and 5.8% at 30 min by the released iron at pH 3 and 5, respectively. Removal of RhB increased with the increasing concentration of released iron based on time. However, degradations were 89.5% and 83.7% at 30 min by schwertmannite at pH 3 and 5, respectively. This significant difference indicates the dominating degradation on the surface of heterogeneous schwertmannite.

### 3.6. Comparison of Heterogeneous Process and Homogeneous Process

**Figure 7:**
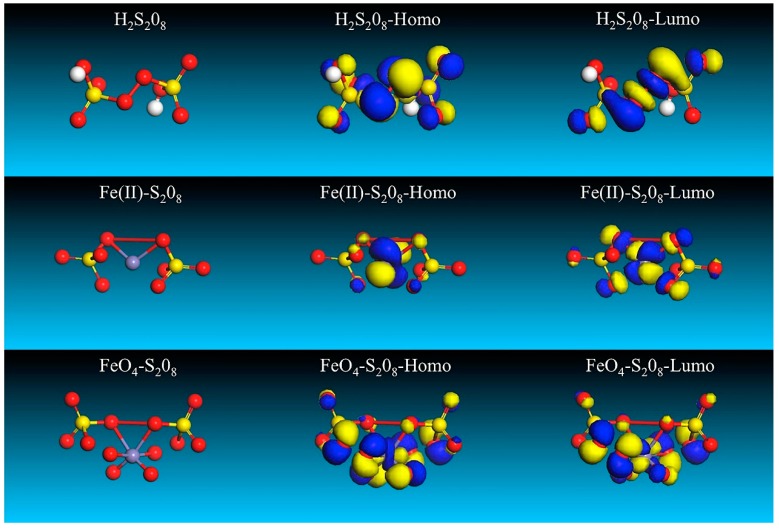
HOMO and LUMO orbital analysis of possible intermediates in homogeneous and heterogeneous process.

To further investigate advantages of heterogeneous process compared with homogeneous process, possible homogeneous and heterogeneous intermediates were provided, as shown in Figure 7, where Fe(II)-S_2_O_8_ and FeO_4_-S_2_O_8_ represent homogeneous and heterogeneous intermediates, respectively. The activation of persulfate would then be considered as the transition of electron from HOMO (Highest Occupied Molecular Orbital) to LUMO (Lowest Unoccupied Molecular Orbital) inside intermediates. Orbital energy analysis of intermediates is listed in Table 1. The energy difference between HOMO and LUMO indicates the reactivity of intermediates, and they are 4.361, 2.678 and 0.404 eV for H_2_S_2_O_8_, Fe(II)-S_2_O_8_ and FeO_4_-S_2_O_8_, respectively. It suggests that the heterogeneous intermediate shows the lowest orbital energy for the electron transition, which means higher reactivity than that of homogeneous intermediate. Therefore, the enhanced activation of persulfate is dominated by heterogeneous process.

### 3.7. Effect of pH_pzc_ on the Heterogeneous Process

**Figure 8:**
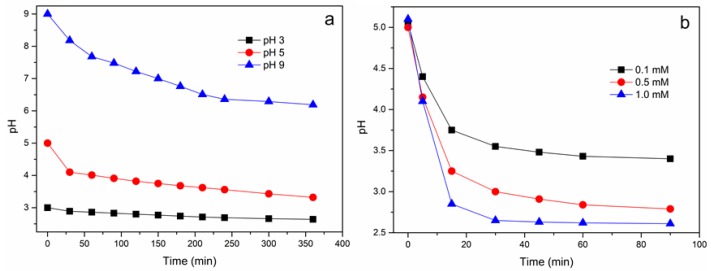
Changes of pH during the RhB degradation in the absence (**a**) and presence (**b**) of HA under various pH conditions. Concentrations: [Sch] = 0.5 g/L, [RhB] = 25 mM, [PS] = 5 mM, and [HA] = 0–1 mM.

The pH point of zero charge (pH_pzc_) of iron oxides catalysts always plays an important role to change the pH in system and provide acidic microenvironment in the heterogeneous AOPs [23,32]. As shown in Figure 8a, the pH decreases from the initial 3, 5 and 9 to the final 2.6, 3.3 and 6.2 in 360 min during the degradation, respectively. Due to the low pH_pzc_ of schwertmannite (3.05) [23], schwertmannite could show the potential to release proton into the environment, where the pH is higher than the pH_pzc_ of iron oxides. The pH of the system would therefore decrease due to the released proton and intermediates formed during the degradation of RhB [23,33,34].

As shown in Figure 8b, the increasing addition of HA finally caused the significant decrease of pH from the initial 5 to the final 3.4, 2.8 and 2.6 with the increasing HA addition of 0.1, 0.5 and 1.0 mM, respectively. Although increasing addition of HA accelerated the transformation from Fe(III) to Fe(II) for the PS activation, it also resulted in the reductive dissolution of schwertmannite during the degradation process. Therefore, the significant decrease of pH is achieved by the release of proton from schwertmannite during the dissolution, and the new-formed acid microenvironment exposed by the dissolution process resulted in the higher degradation rate of RhB.

### 3.8. Activation Mechanism of PS on Schwertmannite in the Presence of HA

**Scheme 1:**
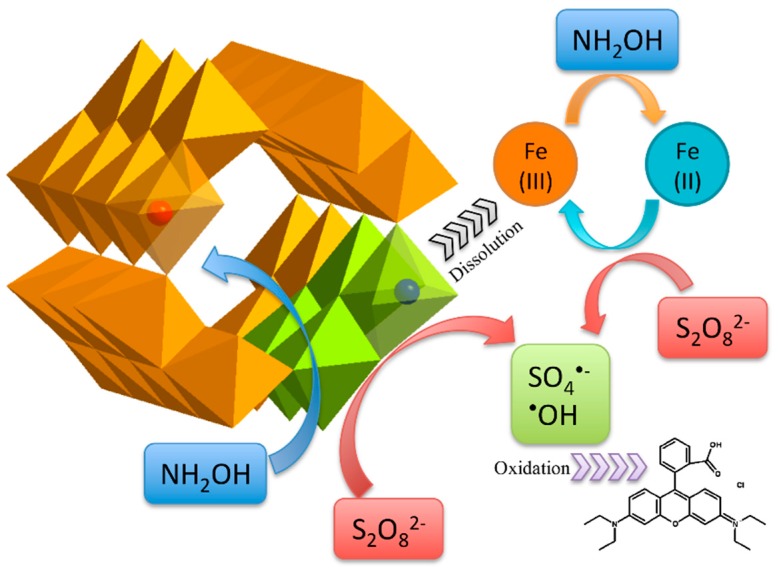
Activation of mechanism of PS on schwertmannite in the presence of HA.

**Figure 9:**
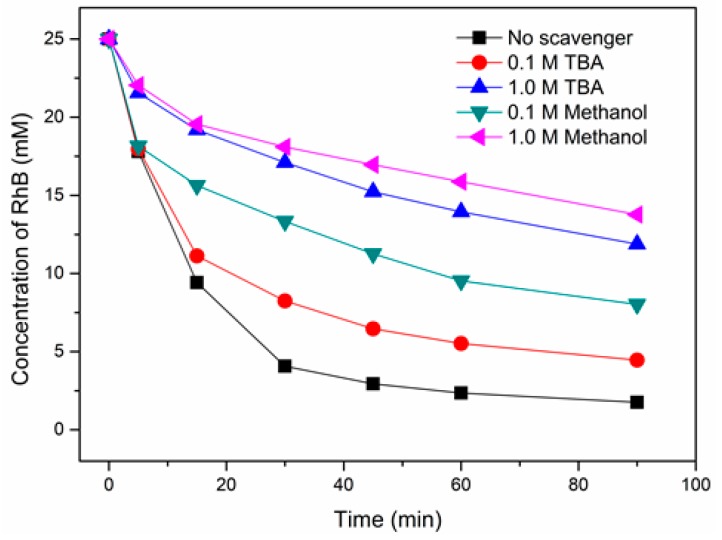
Inhibition effect of radical scavengers on RhB degradation in the presence of HA at pH 5. Concentrations: [Sch] = 0.5 g/L, [RhB] = 25 mM, [PS] = 5 mM, [HA] = 0.5 mM, [TBA] = 0–1 mM, and [Methanol] = 0–1 mM.

To understand the activation mechanism of PS on schwertmannite, methanol and TBA were employed as scavengers for radicals formed by the PS activation. Figure 9 shows the inhibitory effect of methanol and TBA on the degradation of RhB by PS on schwertmannite in the presence of HA. Concentrations of RhB decreased from 25 mM to 8.0 and 13.8 mM with the increasing addition of 0.1 and 1.0 M methanol, respectively. However, the concentrations of RhB decreased to 4.5 and 11.9 mM in the presence of 0.1 and 1.0 M TBA, respectively. Considering the high rate constants for •OH (9.7 × 10^8^ M^−1^·s^−1^) [3] and ${\mathrm{SO}}_{4}^{•-}$ (2.5 × 10^7^ M^−1^·s^−1^) [2], methanol is considered as an effective scavenger for both •OH and ${\mathrm{SO}}_{4}^{•-}$, while TBA is only an effective scavenger for •OH (6.0 × 10^8^ M^−1^·s^−1^) [3] but not for ${\mathrm{SO}}_{4}^{•-}$ (8.0 × 10^5^ M^−1^·s^−1^) [2] due to its much slower rate constant for ${\mathrm{SO}}_{4}^{•-}$. According to the investigation by Zou et al. [15], contribution of ${\mathrm{SO}}_{5}^{•-}$ on the degradation of RhB was excluded. Therefore, the lower efficiency of TBA than methanol for the inhibition of RhB degradation indicates that both •OH and ${\mathrm{SO}}_{4}^{•-}$ are the reactive oxidants that formed by the activation of PS on schwertmannite described as Equations (7)–(12). The possible activation mechanism of PS on schwertmannite in the presence of HA is proposed in Scheme 1.

In strongly acid solutions, persulfate decomposes according to Equations (7) and (8), while, in alkaline, neutral and dilute acid solutions, the decomposition follows Equation (9) [35]. Previous studies suggested that persulfate decomposition in aqueous solutions is first order and that the reaction is catalyzed by hydrogen ion [35,36]. Meanwhile, there are many hydrogen ions on the surface of schwertmannite due to low pH_pzc_ (3.05) [23], which results in the surface of iron oxides existing as the form of Fe-OH_2_^+^ [37]. This contributes to the decomposition of persulfate according to Equations (7) and (8), and then the forming of the Fe-OOSO_3_ peroxo metal complex according to Equations (11) and (12).

(7) $$S_{2}O_{8}^{2-}+H_{2}O\to {\mathrm{SO}}_{5}^{2-}+{\mathrm{SO}}_{4}^{2-}+2H^{+}$$

(8) $${\mathrm{SO}}_{5}^{2-}+H_{2}O\to {\mathrm{SO}}_{4}^{2-}+H_{2}O_{2}$$

(9) $$S_{2}O_{8}^{2-}+H_{2}O\to 2{\mathrm{HSO}}_{4}^{-}+1/2O_{2}$$

(10) $$\equiv \mathrm{Fe}\left(\mathrm{III}\right)+{\mathrm{NH}}_{3}{\mathrm{OH}}^{+}\to \equiv \mathrm{Fe}\left(\mathrm{II}\right)+\mathrm{nitro}-\mathrm{products}$$

(11) $$\equiv \mathrm{Fe}\left(\mathrm{II}\right)-{\mathrm{OH}}_{2}^{+}+S_{2}O_{8}^{2-}\to \equiv \mathrm{Fe}\left(\mathrm{II}\right)-{\mathrm{OOSO}}_{3}^{-}+{\mathrm{SO}}_{4}^{2-}+2H^{+}$$

(12) $$\equiv \mathrm{Fe}\left(\mathrm{III}\right)-{\mathrm{OH}}_{2}^{+}+S_{2}O_{8}^{2-}\to \equiv \mathrm{Fe}\left(\mathrm{III}\right)-{\mathrm{OOSO}}_{3}^{-}+{\mathrm{SO}}_{4}^{2-}+2H^{+}$$

(13) $$\equiv \mathrm{Fe}\left(\mathrm{II}\right)-{\mathrm{OOSO}}_{3}^{-}+H_{2}O\to \equiv \mathrm{Fe}\left(\mathrm{II}\right)-\mathrm{OH}+{\mathrm{SO}}_{4}^{•-}+{\mathrm{OH}}^{-}$$

(14) $$\equiv \mathrm{Fe}\left(\mathrm{II}\right)-{\mathrm{OOSO}}_{3}^{-}+H_{2}O\to \equiv \mathrm{Fe}\left(\mathrm{II}\right)-\mathrm{OH}+•\mathrm{OH}+{\mathrm{SO}}_{4}^{2-}$$

(15) $$\equiv \mathrm{Fe}\left(\mathrm{III}\right)-{\mathrm{OOSO}}_{3}^{-}+H_{2}O\to \equiv \mathrm{Fe}\left(\mathrm{II}\right)+O_{2}+{\mathrm{SO}}_{4}^{2-}+2H^{+}$$

Previous studies suggested that decomposition of H_2_O_2_ on transition metal oxide surface was always achieved by the formation of the peroxo metal complex [23,38,39,40,41]. Similar to the decomposition mechanism of H_2_O_2_ on transition metal oxide surface, the surface hydroxyl group (Fe(III)-OH) on schwertmannite can be substituted by peroxo group (O_3_S-OO-SO_3_) of PS to form Fe(II)-OOSO_3_ or Fe(III)-OOSO_3_ peroxo metal complex (Equations (11) and (12)). After that, the complex decomposes to form radicals (Equations (13)–(15)), and the Fe(II)-OH formed by Equation (10) will transfer to Fe(III)-OH again on the surface for heterogeneous process or aqueous Fe(III) released into solutions for the homogeneous process.

## 4. Conclusions

It was found that the addition of HA was more for the degradation of RhB under acid conditions. Significant release of dissolved iron from schwertmannite was obtained due to the reductive dissolution process. Although the aqueous Fe(II) could be achieved by the involved HA, the transformation from Fe(III) to Fe(II) was accelerated through the reduction of Fe(III)-OH to Fe(II)-OH on the surface of schwertmannite. The newly formed Fe(II)-OH_2_^+^ contributed to the improvement of PS activation for the generation of •OH and ${\mathrm{SO}}_{4}^{•-}$ radicals, which dominated the degradation process.

## Figures and Tables

**Table 1:** Orbital energy analysis of homogeneous and heterogeneous intermediates.

Species	*E*_HOMO_/eV	*E*_LUMO_/eV	Δ*E*/eV
H_2_S_2_O_8_	−11.417	−2.084	9.333
Fe(II)-S_2_O_8_	−11.745	−2.543	9.202
FeO_4_-S_2_O_8_	−9.611	−1.372	8.239
